# Features of Microsystems for Cultivation and Characterization of Stem Cells with the Aim of Regenerative Therapy

**DOI:** 10.1155/2016/6023132

**Published:** 2016-01-31

**Authors:** Kihoon Ahn, Sung-Hwan Kim, Gi-Hun Lee, SeungJin Lee, Yun Seok Heo, Joong Yull Park

**Affiliations:** ^1^School of Mechanical Engineering, College of Engineering, Chung-Ang University, Seoul 06974, Republic of Korea; ^2^Department of Biomedical Engineering, School of Medicine, Keimyung University, Daegu 42601, Republic of Korea

## Abstract

Stem cells have infinite potential for regenerative therapy thanks to their advantageous ability which is differentiable to requisite cell types for recovery and self-renewal. The microsystem has been proved to be more helpful to stem cell studies compared to the traditional methods, relying on its advantageous feature of mimicking* in vivo* cellular environments as well as other profitable features such as minimum sample consumption for analysis and multiprocedures. A wide variety of microsystems were developed for stem cell studies; however, regenerative therapy-targeted applications of microtechnology should be more emphasized and gain more attractions since the regenerative therapy is one of ultimate goals of biologists and bioengineers. In this review, we introduce stem cell researches harnessing well-known microtechniques (microwell, micropattern, and microfluidic channel) in view point of physical principles and how these systems and principles have been implemented appropriately for characterizing stem cells and finding possible regenerative therapies. Biologists may gain information on the principles of microsystems to apply them to find solutions for their current challenges, and engineers may understand limitations of the conventional microsystems and find new chances for further developing practical microsystems. Through the well combination of engineers and biologists, the regenerative therapy-targeted stem cell researches harnessing microtechnology will find better suitable treatments for human disorders.

## 1. Introduction

Stem cells (SCs) are sensitive to various* in vivo* physical/metabolic/biological microenvironmental stimuli and differentiable to necessary cell types [1]. Such multilineage differentiation potential is a promising feature for therapeutic applications to cure of human disorders, such as cardiac disease [2], bone diseases [3], and neurological diseases [4]. The* in vivo* microenvironments of the stem cells are closely linked to intricate cell-friendly spaces with biochemical and mechanical features [5]. However, it is hardly possible to recreate such dynamic and complex cellular environments in traditional dish-based culture [6]. Meanwhile, the biomicrotechniques emerged in the 1990s and have been continuously utilized to develop cell culture microsystems capable of mimicking* in vivo*-like conditions [7] (cell-cell signaling [8], three-dimensional (3D) cell microenvironment [9], and growth factors [10]) and creating engineered stimulations mechanical forces [11, 12] and electrical fields [13].

The most frequently used microtechniques for cell biology study are microwell, micropattern and microchannel. Microwell is micrometer-sized well, which can handle pico- or nanoliters of liquid, for trapping single or multiple cells. The first microwell system was made with poly(methyl methacrylate) (PMMA) in the early 1950s [14], since then microwell techniques have been frequently applied to various biological studies [15]. Comparing the traditional method which used culture dish, using microwell has advantages such as low sample amount, ability of high throughput test, and rapid analysis. Micropattering and microcontact printing are performed by using soft lithography method and used as cell patterning techniques in the past decade. Surface coatings and patterning reform the surface chemistry of a substrate of either cytophilic region or cytophobic region [16]. It can be applied to varied biological research of cellular characterization including control of cell focal adhesion at the microscale [17]. Micropatterning techniques can provide advantages such as dynamic surface properties, ability of various cell-cell interaction, and 3D networks structures [16]. Microfluidic system handles small amount (micro- and nanoliters) of fluids in microscale channel and have been adopted to a lot of applications in multidisciplinary field. Through a lot of studies for several decades [18], the microfluidic system has been believed as an important method with an infinite potential for modern biology research [19, 20]. The microfluidic system, born from microelectromechanical system (MEMS), created a new area of micro total analysis systems (*μ*-TAS) or lab-on-a-chip having the advantages such as small size of device, minimum reagent consumption, quickly reaction time, and most importantly the ability to mimic* in vivo* microenvironment [21]. In this regard, the microfluidic systems have been well appreciated in cell biology researches.

However, the above-mentioned microtechnology-based cell culture systems have been mainly developed by engineers, and thus a gap between the engineers (developer) and the biologists (user) contains issues such as difficulties in understanding design/operation principles, low adaptability, and barriers of the two fields (indifference). For these reasons, though microcell culture systems have been technologically progressed as an important method for cell biology and tissue engineering, these various useful systems do not seem to be well adopted to biologists as principal method [22]. Currently, the enthusiasm and appreciation for the biomicrotechnologies stay mainly in the engineering fields not in the biology field; thus the need is more communication and feedback between the two groups for sharing knowledge of physical/chemical principles in microfluidics and understanding the practical requirements from biologists.

In this review, we will introduce the three major microsystem-based cell culture techniques (microwell, micropattern, and microchannel) which have been used for various analyses about stem cell research. The focus is on explaining specifications of the addressed microsystems regarding physical principles, distinct characteristics, applied stem cell types and revealed stem cell characteristics to reduce the gap of between engineers and biologists in stem cell research. The review is developed as follows: first, the microwell and how to apply it to stem cell studies; second, the micropatterning technique which were focused on the effect of the surface topology on stem cell reaction; third, the physical principle of diffusion/gradient/shear stress created in microfluidic channel and its application.

## 2. Microwells

**Figure 1:**
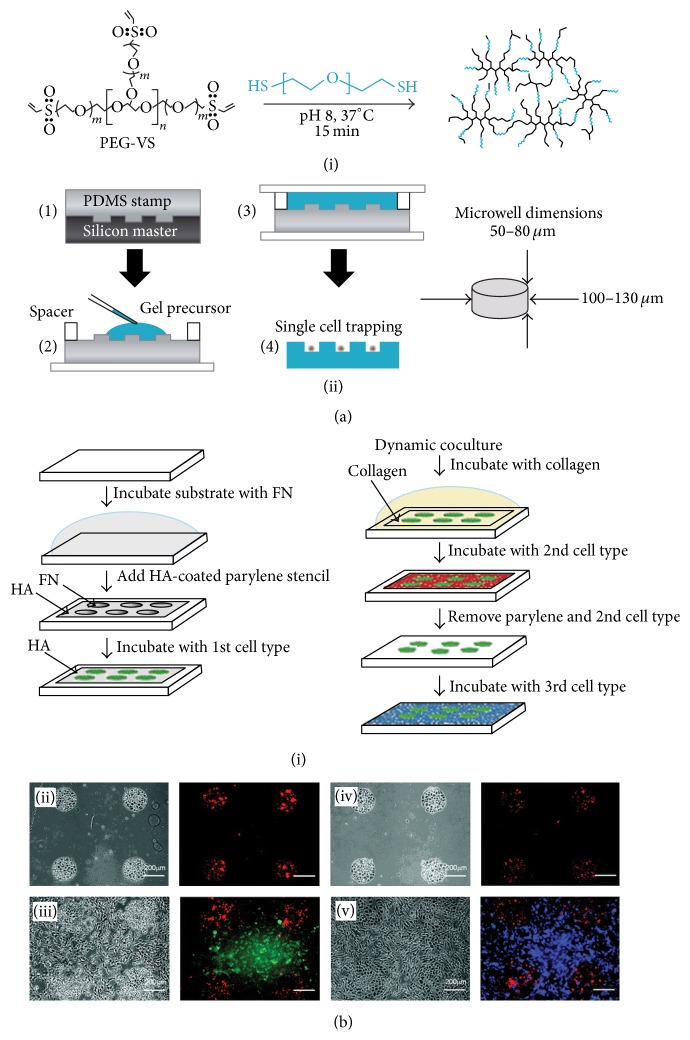
(a) Fabrication of single HSCs microwell array. (i) Reactive thiol and vinyl sulfone end-groups on poly(ethylene glycol) (PEG) precursors. (ii) Overview of hydrogel microwell array fabrication. A PDMS stamp is cast on a silicon master (step 1). This stamp is used as a template to crosslink a PEG gel containing the complementary microwell array topography (steps 2 and 3). Upon swelling and washing, the hydrogel surface is used to trap individual HSCs (step 4). Typical dimensions of microwells are indicated. (b) (i) Coculture system using parylene-C stencils. For cell culture on PDMS, fibronectin was coated on the PDMS surface. (ii)~(v) Light micrograph (*left*) and the corresponding fluorescent (*right*) images of the steps in the formation of dynamic cocultures using parylene-C stencils. (ii) Hyaluronic acid-coated parylene-C stencil was reversibly sealed on fibronectin treated PDMS and seeded with mES cells. (iii) The patterned cocultures of mES cells and AML12 hepatocytes. (iv) To generate dynamic cocultures, the stencil was gently peeled away, leaving the mES cells. (v) After depositing a layer of fibronectin, a third cell type (NIH-3T3) was seeded on the exposed surface. Scale bars are 200 *μ*m.

The weakness of conventional cell culture using a Petri dish or a multiwell dish is that individual cell responses are ignored and only a mix of the bulk responses of a population of cells is represented [23]. For stem cell research, it is important to collect data from isolated individual cells or cell pallets in high-throughput manner, because both isolated cell response and statistical analysis should be obtained simultaneously in order to understand stem cell characteristics clearly. In this regard, microwell array has been used as one of primary pioneering microtechniques [15]. One of examples to apply microwell technique to stem cell research was to recreate niche structure to understand the effects of specific proteins on stem cell function. With hydrogel-based microwell arrays,* in vivo* microenvironment, or niche, is embodied assessing the effects of either secreted or tethered proteins characteristics on hematopoietic stem cell (HSC) fate [24]; 50–80 *μ*m height and 100–130 *μ*m diameter cylindrical-shaped microwell array was fabricated with poly(ethylene glycol) (PEG) (Figure 1(a)). By reactive microcontact printing, protein A site selectively anchors Fc-chimeric proteins on the microwell array which can be artificial niches. HSCs were seeded per well of a 96-well plate; each well contains 400 microwells in 200 *μ*L of medium. After cell seeding, the individual cells were randomly sedimented to the bottoms of the microwells. This study showed that the kinetic behavior of single or rare adult stem cells with simple coculture by tethering properly oriented transmembrane niche proteins. Also the number of cells in microwell can be easily controlled to make desired size of cell pellet. The biomimetic physicochemical properties of the hydrogel substrate could simulate soft and hydrated microenvironment of HSCs in the bone marrow.

Controlling the size or shape of stem cells has been found to regulate cellular fate decisions. Decision of a mesenchymal stromal cell (MSC) to become either a fat or a bone cell depends on the size/shape the cell, which correlated with the activation of the RhoA signalling which is an integrator of structural and soluble cues in developmental processes [25]. For example, hMSC adipogenesis occurred in small group of the cells, osteogenesis occurred only in large group of the cells, and a mixture of both lineages was found on intermediate-sized environment. It is thus possible that microwell size/shape can determine stem cell fate.

The size of ES cell aggregates has been shown to influence lineage specific differentiation in various microwell size (from 40 *μ*m~150 *μ*m) [26]. Shape effect to fate decision has been proved for single cell level and size effect for stem cell pellet. However, more study should be done to clarify the shape effect for stem cell pellet.

A various sizes of hydrogel microwells have been used to determine mouse embryonic stem cell aggregate size effect [27]. In lager embryonic bodies (EBs) formed in larger microwells (450 *μ*m in diameter) cardiogenesis was enhanced, and in contrast, endothelial cell differentiation was increased in smaller microwell (150 *μ*m in diameter). It indicated that EB size could be an important parameter in ES cell fate specification via differential gene expression of members of the noncanonical WNT pathway which is known to be important in cardiogenesis and endothelial cell differentiation.

Microwell function can diversify via surface coating such as matrigel extracellular matrix coating [28]. Culturing on the matrigel-coated microwell (hole sizes: 50, 100 *μ*m, depths: 50, 120 *μ*m), human embryonic stem cells (hESCs) retained pluripotency, differentiating to embryonic germ layers. Similar to microwell coating technique, precoated stencil also can be used. Using parylene-C microstencil, coculture system of embryonic stem (ES) cells was studied [29]; ES cells were cocultured with fibroblasts and hepatocytes by using the reversible sealing stencil on the microwell patterned substrate (range from 40 *μ*m to 200 *μ*m). In this approach, the coculture systems of three type cells are shown in (Figure 1(b)). Since the stencil can be removed in particular time point, ES cells can interact with a defined cell type for a particular period followed by exposure to another cell type.

**Figure 2:**
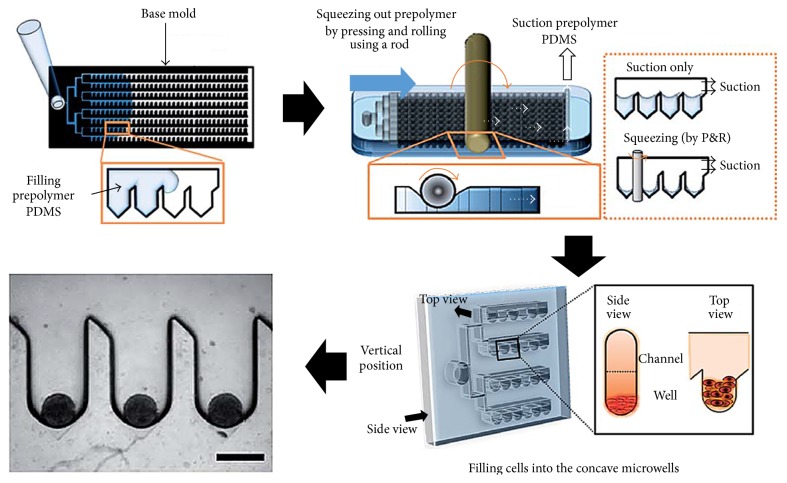
Schematic representation of concave microwell generation utilizing surface tension force. First, the pentagonal-shaped microwells were filled with prepolymer solution and exposed to squeezing/suction. Concave wells were created by a little residual polymer solution due to the surface tension. The ESCs were then injected into microchannel (and microwells) for trapping a uniformal quantity of ESCs to produce the same-sized EBs.

Another microwell was suggested to create uniform-sized EBs using principle of surface tension and capillary force. Surface tension is observed on the liquid-gas interface. In macroscale world, other forces such gravity and inertia are much greater than the surface tension or capillary force. However, surface tension is dominant over the gravitational body forces due to size effect, which can be explained via the scaling laws [30]. More attention from the microfluidic field should be drawn to the dominant forces in microscale world such as surface tension, osmosis, and Van der Waals force, to improve usability and functionality of microfluidic systems used in cell research. The equation of surface tension and pressure variation can be written as Δ*P* = *γ*(1/*R*_1_ + 1/*R*_2_), where *γ* is the liquid surface free energy and *R*_1_ and *R*_2_ are radii of curvature in each of the axes that are parallel to the surface [19]. The capillary force is mainly influenced by surface conditions and device design; therefore, principle of capillary force would be sufficiently considered for the microfluidic device fabrication [31]. For the large quantity production of uniform-sized EBs, many deep concave wells were patterned first and then partially filled up with polymer solution. Due to the surface tension (capillary force), the surface of the polymer solution in each microwell created concave surface which were then cured to serve as 3D curved concave microwell (diameters of 300, 500, and 700 *μ*m). The ES cells were seeded in these concave-bottomed total of 300 microwell array to obtain uniform-sized spherical EBs (Figure 2) [32]. This study is a good example study which showed how the capillary force was cleverly adopted to fabricate microwell systems applied to EB research.

## 3. Micropattern

**Figure 3:**
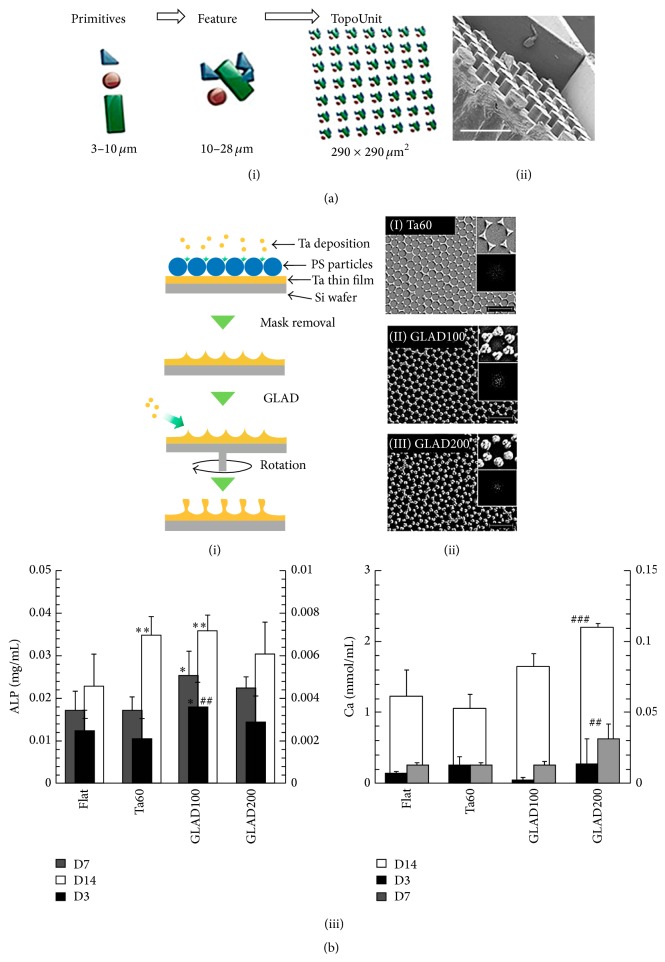
(a) An algorithm-based topographical chip. (i) Fabrication of the topographical chip (poly(lactic acid)) was based on the unique mask design using mixed primitives (circles, triangles, and rectangles). (ii) SEM images of a section of the patterned feature (scale bar: 50 *μ*m). (b) Fabrication of micropillar array with GLAD technique. (i) Fabrication of tantalum (Ta) nanotopographies using a combination of colloidal self-assembly, colloidal lithography, and glancing angle deposition (GLAD) techniques. After assemble 722 nm poly(styrene) colloids, 60 nm Ta (Ta60) was sputtered, and then mask was removed, and GLAD of Ta was done for increasing of feature heights (GLAD100 or GLAD200). (ii) Scanning electron micrographs (SEMs) of Ta60, GLAD100, and GLAD200 (scale bar: 2 *μ*m). (iii) Alkaline phosphatase (ALP) and calcium (Ca) quantification of osteogenic differentiated human adipose derived stem cells (hADSCs) on the surfaces by the cells were quantified with at least three samples (*n* > 3). # and *∗* indicate that there is a significant difference between FLAT and Ta60, respectively.

Micropillar array provides uneven surface to cells and often with particular coating to evoke specific cellular responses, inducing different cellular morphologies [33]. Additionally, micropillar array provides opportunities for mechanical interlocking and suitable attachment matrixes. One example study was done to show the expansion of hematopoietic stem cells on murine stromal cells by 3D micropillar array [34]. The murine fibroblast cell line M2-10B4 is seeded on the collagen coated pillars which have a 50 *μ*m diameter and 85 *μ*m height with lattice spacing of 50 *μ*m. The results showed a significantly higher expansion of HSCs and higher production of cytokines in the micropillar compared to the 25 T flask. Preexisting and common idea of micropillar structures is homogeneous shape such as circular or square columns; however, heterogeneous and designed shapes have been tried for micropillar array system (Figure 3(a)) [33]. Using mathematical algorithms, nonbiased and random 2,176 different topographic surface features were designed by combinations of three types of primitive shapes (circles, triangles, and rectangles). This study shows that the osteogenic differentiation of hMSCs was enhanced by surface topography. The expression of alkaline phosphatase of human mesenchymal stromal cells, a marker for early osteogenic differentiation, was analyzed.

The effect of nanoscale pillar was compared with microscale pillar in terms of mesenchymal stem cell interaction with the pillar-patterned surfaces [35]. According to different physical and topological surfaces (flat, micro-, nanopillars; 2 *μ*m and 20 nm pillars), adipogenic, chondrogenic, and osteogenic differentiation paths were examined to show that nanoscale pillars have greater impacts on mesenchymal stem cell growth, adhesion, lineage specific, and osteogenic differentiation. Another example is that adhesion, proliferation, and osteogenic differentiation of human adipose-derived stem cell were studied on hexagonally close-packed crystal array nanopillar structures [36]. The nanostructure was fabricated using a combination of facile techniques including colloidal self-assembly, colloidal lithography, and glancing angle deposition (GLAD). The fabrication procedure is shown in Figure 3(b). Tantalum deposition, biocompatible material, fills the gap between polystyrene beads, and then, removing the beads, hexagonally close-packed crystal array can be obtained and finally GLAD of Ta increases the structure heights. GLAD technique was used to increase the surface roughness, feature height facilitating extracellular matrix component deposition, and osteogenic differentiation. The osteogenic differentiation of hASCs in terms of alkaline phosphatase activity and calcium deposition was stimulated on GLAD surface.

The poly(vinyl alcohol)- (PVA-) micropatterned polystyrene plate was introduced to mimic the* in vivo* structure and to confirm the influence of between micropattern width and hMSCs differentiation. The surface had twelve different width micropattern stripes (5 to 1000 *μ*m) and hMSCs was differentiated into vascular smooth muscle cells (VSMCs) on each stripe using TGF-beta 1. The results showed that hMSCs is more differentiated into VSMCs in narrow width micropattern stripes (20 to 200 *μ*m) comparing other range of width and nonpatterned region [37].

Another surface-patterning method using polymer film was reported. To generate micropatterned ridge/groove geometries on poly(*ε*-caprolactone) (PCL) film, the flexible film was stretched uniaxially in 54°C. Cultured on the polymer film, MSCs aligned along the ridges with elongated morphology. The geometric cues and mechanical properties were different depending on prefabricated types of PCL film (solvent cast, cast-stretch, and heat-press). The micropatterns regulated MSCs function for tunica media construction. Moreover, MSCs obtained a contractile smooth muscle cells-like phenotype with the expression of the contractile makers [38].

## 4. Microfluidic Channel

Signaling molecules have an important role for specific functions of stem cells. For study about molecular interactions, the microfluidic system can provide great opportunity to effective research for biological functions of stem cells [39, 40]. In microfluidic systems, flows are mostly laminar which has high diffusion and low convection. Laminar flow is diffusion dominant flow which is undisturbed paralleled flow layers where no cross-currents (convective mixing) exist. On the contrary, turbulent flow is convective mixing dominant flow characterized by the random motion of fluid particles. It has been shown that flow changes its stance between laminar and turbulent flows according to the dominance of inertia/momentum effect compared with viscosity effect (or vice versa), which is represented with Reynolds number (= inertial forces versus viscous force; Re = *ρUD*/*μ*, where *ρ* is the fluid density, *U* is the velocity, *D* is the pipe diameter, and *μ* is the fluid viscosity). For a pipe flow, when Reynolds number is less than 2100, it becomes laminar; this value physically means that the viscous effect is comparable to the inertial force. In microscale channels, however, *D* is 10^−3 ^m order, guaranteeing small Reynolds number (thus laminar flow) for most microchannel flows in microfluidic systems [19]. Therefore, convective mixing is much suppressed due to the inherent laminar characteristics mentioned above, and diffusive mixing becomes a dominant phenomenon. Naturally, most microfluidic systems have been designed in consideration of this fundamental fluid dynamic feature for effective operation [41, 42].

### 4.1. Diffusion and Concentration Gradient

Laminar flow is one of the predominant factors in microfluidic systems, and thus the molecular diffusion is the important process. The diffusion is a time-dependent phenomenon. Molecules in the high concentration region diffuse into the low concentration region until an equilibration of concentration is achieved [43]. This process can be explained by Fick's law; *J* = −*D*∂*ϕ*/∂*x*, where *J* is the diffusion flux, *D* is the diffusion coefficient, *ϕ* is the concentration, and *x* is the distance. The microfluidic system can provide an ability for the control of the concentration gradient of biomolecules, cellular microenvironment by using diffusion phenomenon [44]. In laminar flow, mixing of different solutions is achieved by diffusion. According to Fick's law, the diffusion length has relevance to the diffusion coefficient, time, and flow velocity; $\ell \sim \sqrt{Dt}\sim \sqrt{DL/V}$, where *ℓ* is the diffusion length, *t* is the time, *V* is the flow velocity, and *L* is the traveling length [45]. Since this relationship shows that the diffusion length is controlled by the velocity, various concentration profiles can be made in microfluidic systems by controlling the flow velocity in microchannel where the stem cells grow.

**Figure 4:**
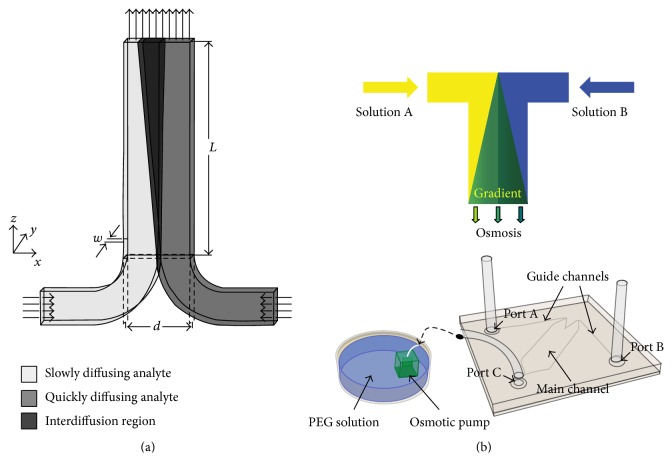
(a) Schematic illustration of the T-channel. Due to laminar flow characteristics, the transportation of molecules between two streams in the channel was induced via diffusion. The two injected sample fluids have different diffusion coefficients; the asymmetry interdiffusion profile is generated in the microchannel. (b) Schematic representation of utilized microfluidic system with osmotic pump. The microchannel and osmotic pump were connected by a flexible tube and the flow is generated by the osmotic pump to generate concentration gradient profile along the microchannel.

T-channel is one of popular microchannel geometries to have two different solutions from each inlet which come into the main channel to create molecules transportation among the two steams only* via* diffusion by utilizing laminar flow characteristics (Figure 4(a)) [46, 47]. The biological and chemical processes are significantly considered about gradient of solutions properties [48, 49]. Many studies have reported that the cells are influenced by concentration gradient of diffusible signalling molecules [50, 51]. To confirm the importance of concentration gradient in biology, many methodological approaches have been developed fabricating specific channel shapes [52–55].

A microfluidic device was introduced to create concentration gradient in a simple Y-channel (width 4 mm, height = 250 *μ*m) where hMSCs were cultured (seeding density of 5 × 10^5^ cells/mL). The flow in this system was driven by a passive (without need of external power) pump called osmotic pump (10 × 10 × 10 mm). This pump provided low and steady flow using osmotic phenomenon between poly(ethylene glycol) solution and DI water. The efficacy of the device was demonstrated by testing hMSCs; the device enabled hMSCs to change properties such as cells viability, attachment, and morphology according to FBS concentration gradient (the attachment of hMSCs is performed in strong concentration of FBS). The advantage of this system includes portability; the device with pumping unit can be easily kept in conventional incubator. This system was also applied to neuronal progenitor cell to study the influence of cytokine concentration (Shh and FGF8 versus BMP4) on neural development (differentiated into neurons). Initially the hESCs-derived neural progenitor cells were seeded (density of 5 × 10^6^ cells/mL) onto channels. The osmosis pump provided extremely slow flow rate that is enough to create the cytokines gradient concentration and to minimize shear effects to the cells (Figure 4(b)) [56, 57].

**Figure 5:**
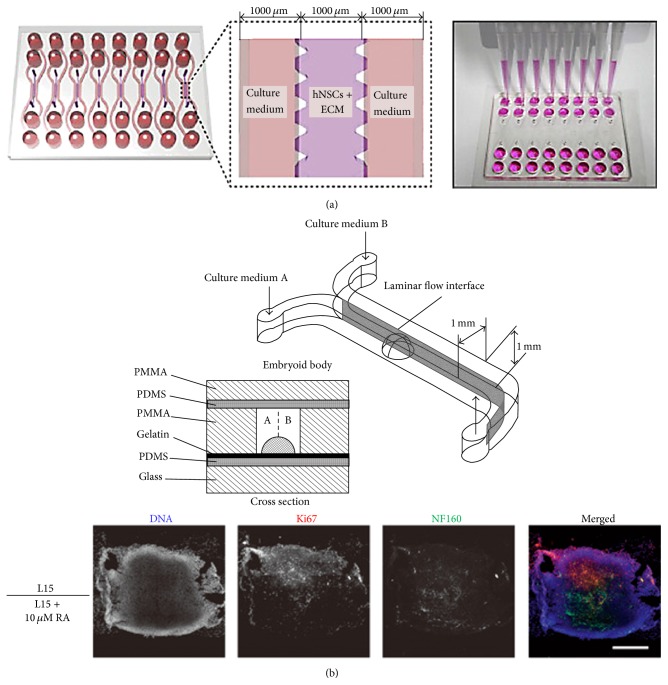
(a) Schematic illustration of niche-like microsystem. The central culture chamber was seeded with hNSCs with hydrogel solutions and the two sides of channels were filled with culture medium. Therefore, the needed molecules were provided to the hNSCs through the diffusion from the side channels. (b) Schematic representation of microfluidic system for EB differentiation. Two different solutions (one with growth factor) were injected to the Y-channel. The advection became more dominant than diffusion preventing growth of concentration gradient profile; therefore, the differentiation was developed only one side of the EB. Image analysis confirmed the influence of 10 *μ*M RA (retinoic acid).

To mimic* in vivo*-like 3D microenvironment for human neural stem cell (hNSCs), a microfluidic channel array was introduced; this system consists of central channel for cell culture and two side channels for flowing culture media. The hNSCs were loaded into the extracellular matrix hydrogel solutions (COL I, Col I + FN, Col I + LN, and Col I + FN + LN) and the cell/hydrogel mixture was injected into the central channel for 3D culture (seeding density is 5 × 10^6^ cells/mL). Thus, molecular transport was generated between central cell culture channel and two-side culture media supply channels by diffusion. The hNSCs differentiation was confirmed in normoxia and hypoxia conditions (Figure 5(a)) [58].

Concentration gradient in microchannel is generated by diffusion mechanism since convective mixing is suppressed in low Reynolds number (laminar flow). Therefore, it is a favorable feature of microfluidic gradient generation systems for cell biology application because concentration gradient is biological norm in human body. However, sometimes generation of stratified concentration profiles is needed to test cell characteristics. In this regard, a good example system was introduced to study differentiation of EB cells in stratified biological molecular concentration. To generate stratified flow, a simple Y-channel geometry (1 mm width × 1 mm height) was used and two different types of medium (L-15 culture medium versus L-15 with 10 *μ*M retinoic acid) were injected to the inlets (at the flow rate of 50 *μ*L/min). A single EB was located at the middle of microchannel. This system provides the advection-dominant (compared to the diffusion strength) flow to prevent the mixing of the media, resulting in the fact that only the half of EBs (L-15 with 10 *μ*M retinoic acid for growth factor) were differentiated (Figure 5(b)) [59].

### 4.2. Shear Stress

Relationship between shear stress and cells has been studied in microfluidic systems. Various stem cells including ESCs and MSCs were influenced by the shear stress regarding adhesion and differentiation [60–62]. The shear stress is one of mechanical forces applicable to cell mechanics studies and depends on the velocity gradient at the surface [63]. In most thin microchannels, with the assumptions of laminar/incompressible flow and Newtonian fluid (stress is linearly proportional to the rate of change of its deformation over time [64]), the shear stress can be written as *τ* = 6*μQ*/*h* ^2^ *w* for a rectangular cross section with *w* ≫ *h*, where *τ* is the shear stress, *μ* is the fluid viscosity, *Q* is the flow rate, *h* is the channel's height, and *w* is the channel's width [65]. Although most flow generated in microsystems is laminar flow [66], the effect of the shear stress on cells has been overlooked in most previously reported microsystems. Thus thorough consideration on the influence of shear stress to cells should be done at the design step of the systems and also at data analysis step [9].

**Figure 6:**
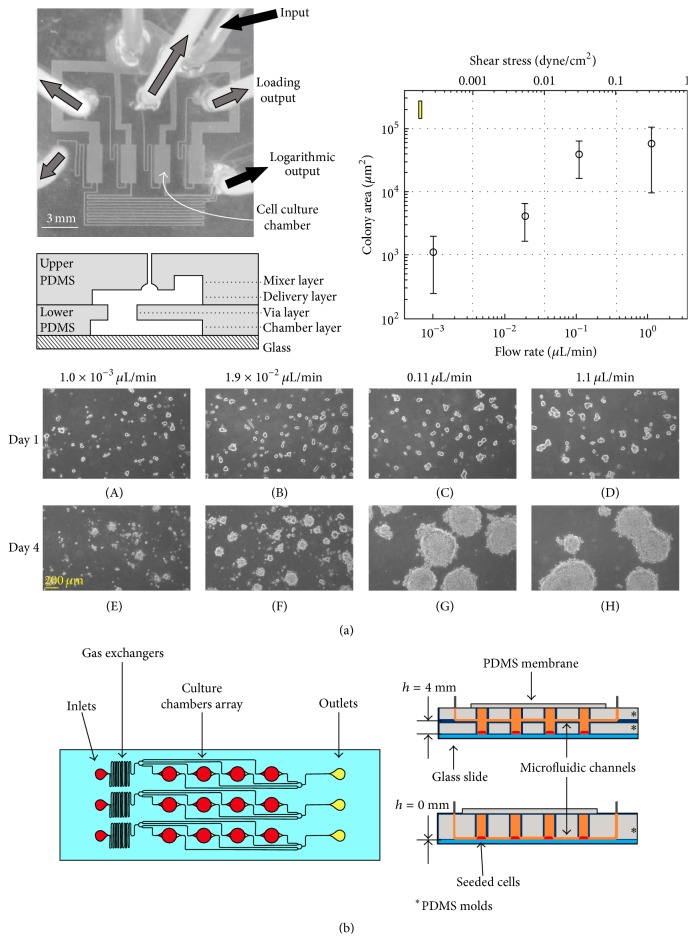
(a) Photograph and schematic illustrate the shear generation device. The microchannel consisted of mixer, delivery, via, and chamber layers. The flow was delivered through inlet; each chamber had different flow rate (shear stress) because of different flow resistance which was induced by the geometry. The data analysis showed that ESC colony development was proportional to the shear stress level. (b) Schematic illustration of another shear generation microfluidic system with gas exchangers. The two types of channels (*h* = 0 mm, *h* = 4 mm) were used to create different shear stress levels. The result showed that the hESCs were more differentiated in high-shear (when *h* = 0 mm).

To investigate how shear stress is influential to morphology of ESC colony, microfluidic device was utilized to control wide range of flow rates. The device has multiple (4 and 16) cell culture chambers with computerized flow resistance controller; ESC colony in each chamber experiences different shear stress levels. This study confirmed that the ESCs colony area increased in the higher shear stresses (about >10^−2^ dyne/cm^2^) (Figure 6(a)) [67]. Another relationship between shear stress and the hESC morphology and differentiation have been researched utilizing microfluidic bioreactor which generates both shear stress and concentration gradient. This system consisted of gas exchanger and culture chamber array (4 × 3 chambers). The hESCs were cultured in each chamber (3.5 mm × 3.5 mm × 100 *μ*m) coated with collagen IV. The results indicate that vascular differentiation increased under higher shear stress level obtained in microfluidic channel (Figure 6(b)) [63]. The hESCs-Human Foreskin Fibroblasts (HFF) coculture study utilizing “flow-stop” system was introduced. The microreactors were designed with consideration of mass transport and shear stress. The hESCs are sensitive to shear stress; the hESCs were usually washed out even under the low shear stress (<0.01 dyne/cm^2^). To remedy this problem, the “flow-stop” operation scheme was adopted in this system; flow cycles of 2 h stop and 2 min flow was repeatedly applied. The hESCs were able to survive under the flow-stop operation in microchannel chambers (10 mm × 20 mm × 100 *μ*m) to prove that the “flow-stop” method was useful for long-term culture of hESC cells [65].

## 5. Summary

Microsystem techniques can be categorized into three different types of techniques. First, microwell is the technique which allows cells to create* in vivo*-like 3D structure. Second, micropatterning can confirm the cells reaction by controlling the micro-/nanoscaled surface topology. Finally, microfluidic channel provides dynamic and precise control of mechanical and biochemical cues such as shear stress and biochemical concentration.  Table 1 summarizes the related operational principles, target cell types, characteristic of system, performance, and regenerative therapy of these methods.

## 6. Conclusions

In this review, various microsystems for stem cell researches were introduced in terms of physical principles (structure, diffusion, shear stress, etc.). For about 20 years, the microsystem techniques have been rapidly progressed, and a lot of useful microsystems were developed for stem cell research; however, only a few of the existing microsystems have been utilized by biologists or clinicians in practices. The mainly reason for this happening might be the gap of view point and background knowledge between engineering and biology fields. The newly developed microsystems are usually and mostly reported in the engineering societies [22], and invisible but strong barrier prevents communications to the people in the biology field. Through this review, we want to provide useful information regarding the microtechniques and applied principles to help understanding for biologists. We also want engineers to be aware of the gap and the need of conditions for expanding microsystems to practical biology labs; the new microsystems should be able to merge into the existing biolaboratory processes, easy to control, and available to be merged with the other laboratory equipment such as microscope and incubator. Also the challenges for both the engineers and biologists include that many other combinations of physical, chemical, and biological principles should be tested and tried to find better way to reveal the stem cell characteristics and control their functions in the future.

## Figures and Tables

**Table 1:** Summarization of the cell operational principles, target cells, characteristics, performance, and regenerative therapy.

Methods	Operational principles	Target cell	Characteristics	Performance	Regenerative therapy	Reference
Microwell	Biochemical reaction	Hematopoietic stem cell (HSCs)	Hydrogel	Proteins characteristic	—	[24]
		Human embryonic stem cell (hESCs)	Hydrogel	Differentiation	Cardiac	[27]
		Human embryonic stem cell (hESCs)	Matrigel	Undifferentiation	—	[28]
		Embryonic stem cells (ESCs)	Parylene-C stencil	Coculture	—	[29]
		Embryonic stem cells (ESCs)	Pressing/rolling	Uniform-sized spheroid	Cardiac	[32]

Micropattern	Surface topology	Hematopoietic stem cell (HSCs)	Micropillar	Cell adhesion/expansion	—	[34]
		Human mesenchymal stem cell (hMSCs)	Micropillar	Differentiation	Osteogenic	[33]
		Mesenchymal stem cell (MSCs)	Micro/nanopillar	Differentiation	Osteogenic	[35]
		Human adipose-derived stem cell (hASCs)	Nanopillar	Differentiation	Osteogenic	[36]
		Human mesenchymal stem cell (hMSCs)	PVA pattern	Differentiation	Vascular	[37]
		Mesenchymal stem cells (MSCs)	PCL film	Differentiation	Vascular	[38]

Microfluidic channel	Diffusion gradient	Human mesenchymal stem cells (hMSCs)	Osmotic pump/Y-channel	FBS effects on hESCs	—	[56]
		hESCs-derived neural progenitor cells	Osmotic pump/T-channel	Differentiation	Neurons	[57]
		Human neural stem cell (hNSCs)	Hydrogel	Differentiation	Neurons	[58]
		Embryonic stem cells (ESCs)	Peristaltic pump/Y-channel	Differentiation	Neurons	[59]
					
	Shear stress	Embryonic stem cells (ESCs)	Syringe pump/valves	Cells morphology	—	[67]
		Human embryonic stem cell (hESCs)	Syringe pump/hydrogel	Differentiation	Vascular	[63]
		Human embryonic stem cell (hESCs)	Syringe pump	Coculture	—	[65]
